# Protocol for the synthesis of N-Alkyl bromomaleimide linkers

**DOI:** 10.1016/j.mex.2026.103809

**Published:** 2026-01-29

**Authors:** Jessica T. Mhlongo, Anamika Sharma, Fernando Albericio, Beatriz G. de la Torre

**Affiliations:** a School of Laboratory Medicine and Medical Sciences, College of Health Sciences, University of KwaZulu-Natal, Durban, South Africa; b Peptide Science Laboratory, School of Chemistry and Physics, University of KwaZulu-Natal, Durban, South Africa; c Department of Inorganic and Organic Chemistry, University of Barcelona, Barcelona, Spain

**Keywords:** Bromomaleimides, Amines, Acetic anhydride

## Abstract

Herein a straightforward and efficient method for synthesizing bromomaleimides with varied carbon chain lengths to modulate molecular properties has been reported. Compounds **1–4** incorporate a single bromomaleimide unit, while compound **5** features two, enabling dual functionalization. This synthetic approach consistently yields high-purity products in good to excellent yields, with compound **5** offering potential for dual payload conjugation due to its bifunctional structure. The key highlights are:

1. Bromomaleimides offers exceptional stability compared to maleimides counterparts

2. Highly selective for Cysteine modification

## Graphical abstract

**Figure:**
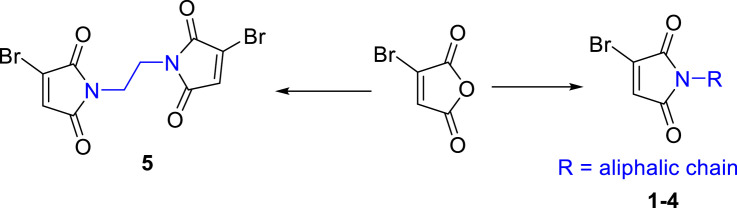

## Specifications table

This table provides general information on your protocol.

**Table:** 

Subject area	Chemistry
**More specific subject area**	Linker chemistry
**Name of your protocol**	Bromomaleimide Synthesis
**Reagents/tools**	**Materials and Reagents**Following chemicals and reagents were required for the synthesis:Bromomaleic anhydride6-Aminohexanoic acid10-Aminodecanoic acidButylamineDodecyl amineEthylenediamineAcetic anhydrideGlacial acetic acidSolvent (ethyl acetate, n-hexane)Cotton woolGlassware (Round bottom flask, reflux condensers, beakers, conical flask, and column)Silica gel for column chromatographySilica-gel coated aluminium Thin Layer Chromatography (TLC) plates**Instrumentation** • Hot plate was used for carrying out the reaction. • Follow-up of the reactions and checks of the purity of the compound were done by TLC on silica-gel-protected aluminium sheets 60 F254 (Merck), and the spots were detected by exposure to UV light at λ = 254 nm. • Rotary evaporator (Buchi, Germany) under pressure was used for removal of solvents. • Compounds were analysed for purity by Shimadzu HPLC using a Phenomenex AerisTMC18 (3.6 µm, 4.6 × 150 mm) column, with a flow rate of 1.0 mL/min and UV detection at 220 nm. 2 µL of each injected on a reversed-phase C18 column (4.6 × 150 mm, 5 µm) operating at 1.0 mL/min, with linear gradients of 0.1 % TFA in MilliQ water and 0.1 % TFA in CH_3_CN as eluents. Data processing was performed using Lab solution software. • Liquid Chromatography-Mass Spectrometry (LCMS) analysis was conducted on an Ultimate™ 3000, AerisTM (Thermo Fisher Scientific, Waltham, MA, USA) with a Phenomenex C18 column (3.9 × 150 mm, 5 µm), a flow rate of 1.0 mL/min and UV detection of 220 nm, with a linear gradient of 0.1 % formic acid in MilliQ water and 0.1 % formic acid in CH_3_CN as eluents. • Magnetic resonance spectra (^1^H and ^13^C) were recorded with Bruker 400 MHz, and chemical shift values are reported in δ units (ppm) using TMS as internal standard.
**Experimental design**	Bromomaleimides were prepared by following the reported protocol in two steps. In the first step, bromomaleic anhydride (1 eq) was refluxed with respective amine (1 eq) in glacial acetic acid as solvent for 8 h, followed by second step by refluxing the reaction mixture for 2 h further by addition of acetic anhydride. The solvent was removed, and the crude was purified by silica gel column chromatography to afford pure products.
**Trial registration**	None
**Ethics**	None
**Value of the Protocol**	1. Bromomaleimides offers exceptional stability compared to maleimides counterparts 2. Highly selective for Cysteine modification

## Background

Modern chemical biology is based on the conjugation of (bio)molecules or the modification of proteins. This has been translated into several antibody-drug conjugates (ADCs) in the market and more ADCs and peptide-drug conjugates in preclinical or clinical phases [1,2]. The key to this strategy is the linker, which is the chemical moiety that binds the (bio)molecules through a Michael addition of the thiol component [3]. There are several linkers, but maleimides are, without doubt, the most used [4]. Maleimides are commonly used for their high selectivity [5,6] and high reactivity towards thiols, in general cysteine [7,8]. Although they have been effectively employed for many years as linkers on ADCs or by the chemical modification of proteins, their use comes with certain limitations, including the reversible nature of the reaction, and, hence, the modification and the number of attachment positions when proteins are modified [9]. Thus, thioethers formed via maleimide conjugation can suffer retro-Michael addition, rendering the free thiol, and limiting their scope [10].

Bromomaleimides feature a bromine atom replacing a hydrogen on the maleimide double bond, enhancing nucleophilicity and enabling further chemical modifications. They are reported to be highly selective and reversible for cysteine modification, as demonstrated by illustration on the SH2 domain of the Grb2 adaptor protein (L111C) [11]. Thus, adding a second thiol to achieve further bioconjugation demonstrates that bromomaleimides offer opportunities for up to three points of attachment [12]. The resultant protein-maleimide products can be cleaved to regenerate the unmodified protein, proving that the reaction can be irreversible [13]. Bromomaleimides can also act as Michael acceptors, reacting fast (<1 min in H_2_O/DMF) with cysteines to produce thiomaleimides adducts [11]. Therefore, bromomaleimides can be used as a tunable reagent for bioconjugation of biomolecules [14,15]. Bromomaleimides conjugates with three points of chemical attachment can now be reversible or irreversible, stabilized and cleaved in aqueous, thiol-mediated reducing environment by the control of maleimide hydrolysis. Such control of stability allows the synthesis of a range of complex bioconjugates with significantly different potential [16]. Bromomaleimides have also been used as intermediates for drug-like molecules, disulfide rebridging [17] targeted drug delivery, antibody-drug conjugates (ADCs) [18], protein labeling with fluorophores or affinity tags enzyme activity modulation [19]. They are used in smart materials and functional polymers due to their reactivity and ability to form cross-links [20]. The reversible thiol‑bromo substitution allows for self-healing materials and reprocessable thermosets [21,22].

Recently, Iris Biotech has synthesized “next generation maleimides” (NGMs) [23], which comprise a set of brominated maleimides and pyridazinediones (PDs) that readily react with the thiols of cysteines. After the formation of the unsaturated thioether(s), the maleimide ring is hydrolyzed at pH 8 to yield a maleamic acid which stabilizes the thioether(s). Like RL-8795 and RL-8815, have been shown to conjugate to cysteine within 1 min, and they hydrolyze quantitatively after 1 hour. This reaction saves time and minimizes the risk of potential damage that can happen to the conjugate from the common alkaline buffer conditions which are usually required for bromomaleimide hydrolysis. Also, the resulting conjugate are unluckily to be degraded before they reach lysosomes since they are stable at physiological pH and at pH 5.5 (endosomes physiological conditions) [23].

Our group has reported that bromomaleimide can be used as a connector to synthesize branched peptides [12,24]. We report herein synthesis of the N-functionalized bromomaleimides, which are easily synthesized with readily available and cost-effective reagents/solvents, clean and short synthesis and obtaining high yield pure product. The length in the N-alkyl maleimide has been varied affording **1–5**. Increasing the N-alkyl chain length of maleimides enhances hydrolytic stability through a combination of inductive electron donation and reduced local solvation of the imide carbonyls. As inductive effects saturate rapidly with increasing chain, steric and hydrophobic shielding continue to suppress hydrolysis without significantly impairing thiol reactivity. Thus, the products are usually solid and stable at room temperature (25 °C), allowing them to have better shelf life.

## Description of protocol

### General synthetic protocol

Respective amine (1.5 mmol) was dissolved in 25 mL glacial acetic acid in a round bottom flask while stirring on a hot plate. Bromomaleic anhydride (1.5 mmol for **1–4** and 3.0 mmol for **5**) was added dropwise, and the reaction was refluxed for 8 h. After completion (as monitored by TLC for consumption of starting material), reaction mixture was cooled to room temperature. Acetic anhydride (2 mL) was then added to the reaction mixture and refluxed further for 2 h. Acetic acid was removed using a rotatory evaporator under reduced pressure. The crude was then purified using silica gel column chromatography using ethyl acetate and n-hexane as the mobile phase. In case of butyl amine, dodecyl amine, hexanoic acid, decanoic acid, and ethylenediamine, the desired product elutes at 25 %, 1 %, 14 %, 5 % and 3 % ethyl acetate in n-hexane, respectively. The products were collected until no corresponding spot were seen on the TLC. The collected fractions were concentrated under reduced pressure on the rotary evaporator. All the products were obtained in high yields and purity.

#### 3‑bromo-1‑butyl‑1H-pyrrole-2,5‑dione (1)

**Yield** = 320 mg (92 %); **State** = Solid; **^1^H NMR (600** **MHz, CDCl_3_)**: δ 6.87 (s, 1H, CH), 3.57 (t, J = 14.6 Hz, 2H, CH_2_), 1.60–1.55 (m, 2H, CH_2_), 1.34–1.28 (m, 2H, CH_2_), 0.93 (t, J = 7.4 Hz, 3H, CH_3_) ppm; **^13^C NMR (150** **MHz, CDCl_3_)**: δ 13.5, 19.9, 30.5, 38.7, 131.3, 131.8, 165.4, 168.6 ppm; **HRMS (APCI) m/z**: Compound did not ionize.

#### **3‑bromo-1-dodecyl-1H-pyrrole-2,5‑dione** (**2**)

**Yield** = 465 mg (90 %); **State** = Solid; **^1^H NMR (600** **MHz, CDCl_3_)**: δ 6.86 (s, 1H, CH), 3.55 (t, J = 14.6 Hz, 2H, CH_2_), 1.61–1.57 (m, 2H, CH_2_), 1.30–1.25 (m, 18H, CH_2_), 0.88 (t, J = 7.0 Hz, 3H, CH_3_) ppm; **^13^C NMR (150** **MHz, CDCl_3_)**: δ 14.1, 22.7, 26.7, 28.5, 29.1, 29.3, 29.4, 29.5, 29.6, 31.9, 39.0, 131.3, 131.8, 165.4, 168.6 ppm; **HRMS (APCI) m/z**: Compound did not ionize.

#### **6-(3‑bromo-2,5-dioxo-2,5-dihydro-1H-pyrrol-1-yl)hexanoic acid** (**3**)

**Yield** = 387 mg (89 %); **State** = Solid; **^1^H NMR (600** **MHz, CDCl_3_)**: δ 6.87 (s, 1H, CH), 3.57 (t, J = 7.2 Hz, 2H, CH_2_), 2.36 (t, J = 7.4 Hz, 2H, CH_2_), 1.69–1.60 (m, 4H, CH_2_), 1.38–1.32 (m, 2H, CH_2_) ppm; **^13^C NMR (150** **MHz, CDCl_3_)**: δ 24.0, 26.1, 28.1, 33.7, 38.6, 131.3, 131.8, 165.3, 168.6, 179.3 ppm; **HRMS (APCI) m/z**: Calcd for C_10_H_13_BrNO_4_: 290.0028 [M + H]+; found 290.0017.

#### **10-(3‑bromo-2,5-dioxo-2,5-dihydro-1H-pyrrol-1-yl)decanoic acid** (**4**)

**Yield** = 467 mg (90 %); **State** = Solid; **^1^H NMR (600** **MHz, CDCl_3_)**: δ 6.86 (s, 1H, CH), 3.56–3.54 (m, 2H, CH_2_), 2.36–2.34 (m, 2H, CH_2_), 1.64–1.58 (m, 2H, CH_2_), 1.36–1.26 (m, 12H, CH_2_) ppm; **^13^C NMR (150** **MHz, CDCl_3_)**: δ 24.6, 26.6, 28.4, 29.0, 29.1, 29.2, 33.9, 38.9, 131.3, 131.8, 165.4, 168.7, 179.2 ppm; **HRMS (APCI) m/z**: Calcd for C_14_H_21_BrNO_4_: 346.0654 [M + H]+; found 346.0652.

#### **1,1′-(ethane-1,2-diyl)bis(3‑bromo-1H-pyrrole-2,5‑dione)** (**5**)

**Yield** = 482 mg (85 %); **State** = Solid; **^1^H NMR (600** **MHz, CDCl_3_)**: δ 7.50 (s, 1H, CH), 7.49 (s, 1H, CH), 3.62–3.61 (m, 4H, CH_2_) ppm; **^13^C NMR (150** **MHz, CDCl_3_)**: δ 37.1, 130.9, 131.1, 165.9, 169.2 ppm; **HRMS (APCI) m/z**: Calcd for C_10_H_7_Br_2_N_2_O_4_: 376.8773 [M + H]+; found 376.8758.

## Protocol validation

**Scheme 1:**
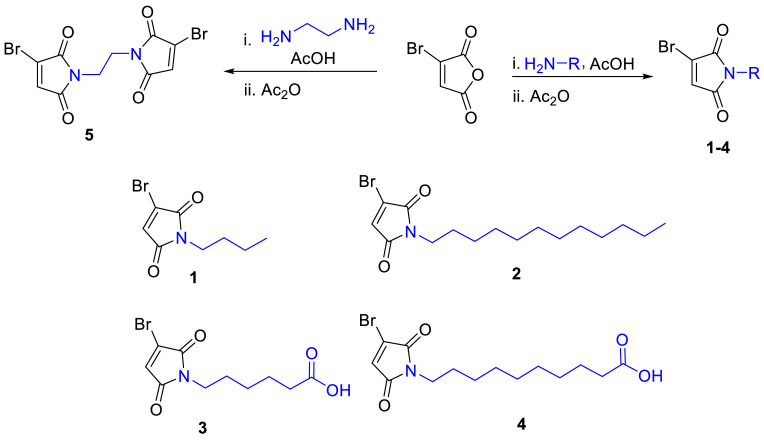
Synthetic protocol for the synthesis of bromomaleimides.

Bromomaleimides were prepared by following the reported protocol in two steps [12,25]. In the first step, bromomaleic anhydride (1 eq) was refluxed with respective amine (1 eq) in glacial acetic acid as solvent for 8 h, followed by second step by refluxing the reaction mixture for 2 h further by addition of acetic anhydride (Scheme 1). However, in the reported literature the reaction was heated to 140 °C for longer hours followed by washing of crude using toluene before silica gel column purification [26,27]. However, in our case, the reaction was refluxed for much less time (3 h and then 2 h). The solvent was removed, and the crude was purified by silica gel column chromatography to afford pure products (**1–4**). In case of **5**, 2 eq of bromomaleic anhydride was used with ethylene diamine (1 eq) for incorporation at both amines. The remaining procedure remains as explained above.

The pure products were well characterized by HPLC for purity, and identity by mass and NMR (^1^H and ^13^C). The compounds **1** and **3** were compared with the earlier reports. It was found that the yields in our case was much higher compared to that of the reported literature [26,27]. Derivatives containing fatty acid chains (**1** and **2**) did not ionize for mass confirmation. However the product was confirmed by ^1^H NMR and ^13^C NMR. The spectra are provided in the supplementary information.

## Limitations

None

## Related research article

None

## CRediT authorship contribution statement

**Jessica T. Mhlongo:** Methodology, Formal analysis. **Anamika Sharma:** Writing – review & editing, Writing – original draft, Conceptualization. **Fernando Albericio:** Writing – review & editing, Validation, Supervision, Funding acquisition, Formal analysis, Conceptualization. **Beatriz G. de la Torre:** Writing – review & editing, Validation, Supervision, Funding acquisition, Formal analysis, Conceptualization.

## Declaration of competing interest

The authors declare that they have no known competing financial interests or personal relationships that could have appeared to influence the work reported in this paper.

## Data Availability

Data will be made available on request.

## Appendix Supplementary materials

Supplementary material and/or additional information [OPTIONAL] The supplementary Information containing the characterization data (HPLC, HRMS, ^1^H NMR and ^13^C NMR) is available free of charge. Image, application 1
