# Supplementary material for: Protocol for the synthesis of N-Alkyl bromomaleimide linkers
Source: MethodsX. 2026 Jan 29;16:103809. doi: 10.1016/j.mex.2026.103809 (PMC12906098; doi:10.1016/j.mex.2026.103809)

**Protocol for the synthesis of Bromomaleimide linkers**

Jessica T. Mlongo,^1^ Anamika Sharma,^2,^* Fernando Albericio,^2,3^ Beatriz G. de la Torre^1,^*

^1^School of Laboratory Medicine and Medical Sciences, College of Health Sciences, University of KwaZulu-Natal, Durban, South Africa

^2^Peptide Science Laboratory, School of Chemistry and Physics, University of KwaZulu-Natal, Durban, South Africa

^3^Department of Inorganic and Organic Chemistry, University of Barcelona, Barcelona, Spain

**Corresponding authors:** SharmaA@ukzn.ac.za; Garciadelatorreb@ukzn.ac.za

***Supplementary Information***

**S1**. HPLC for **1** (HPLC method: 5-95% B into A in 15 min at 220 nm)

**
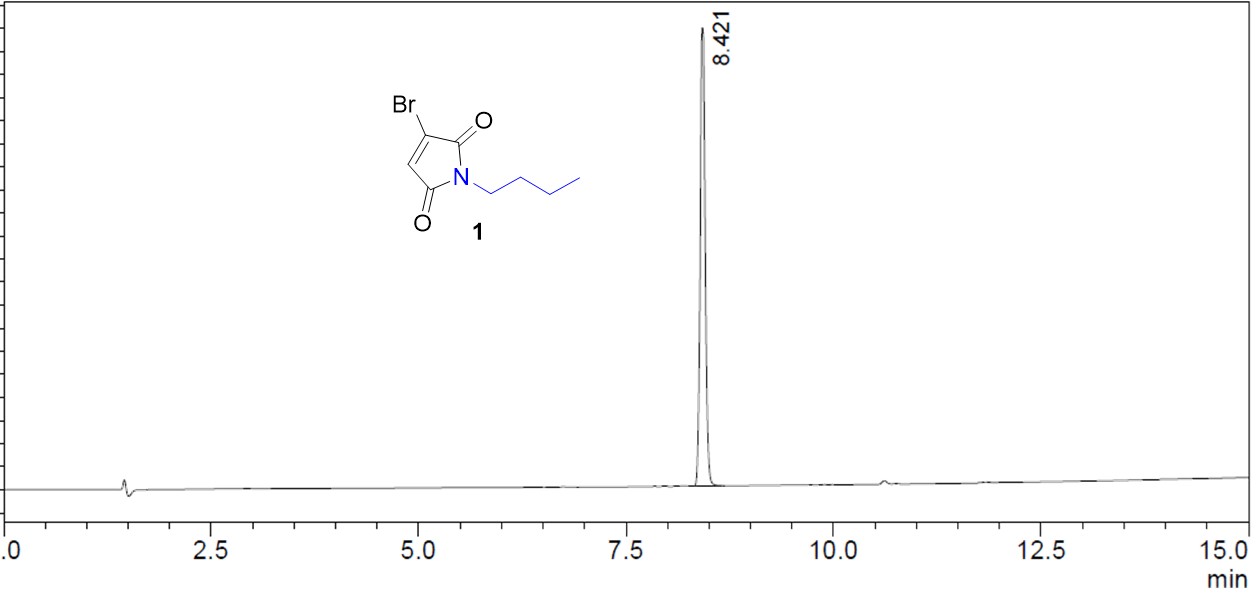
**

**S2.** ^1^H NMR for **1**

**
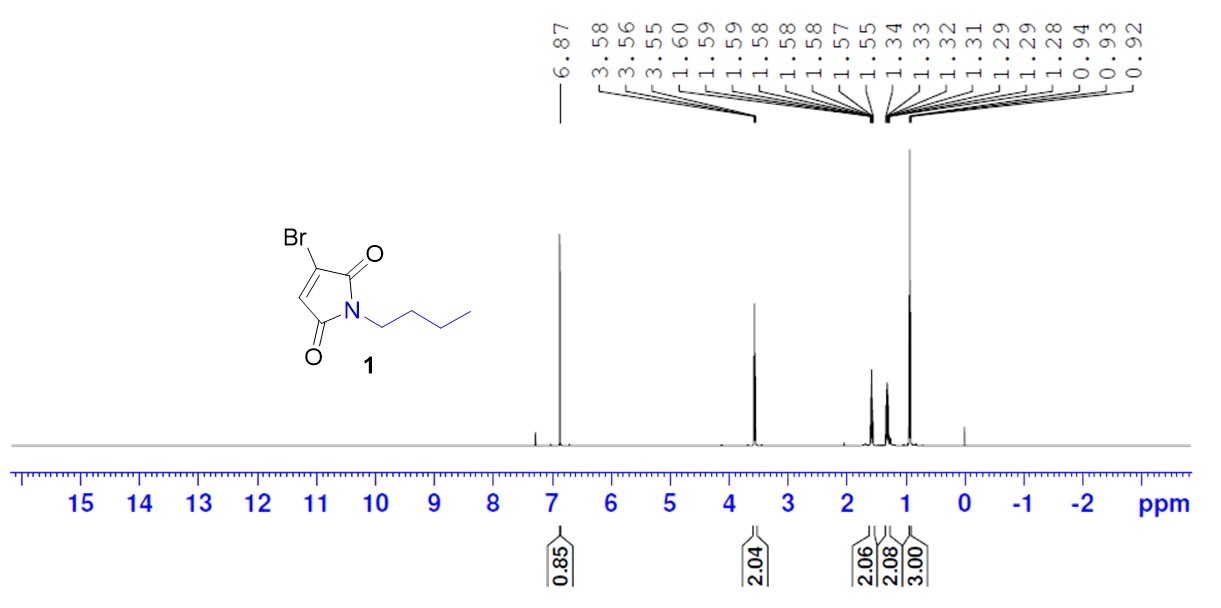
**

**S3.** ^13^C NMR for **1**

**
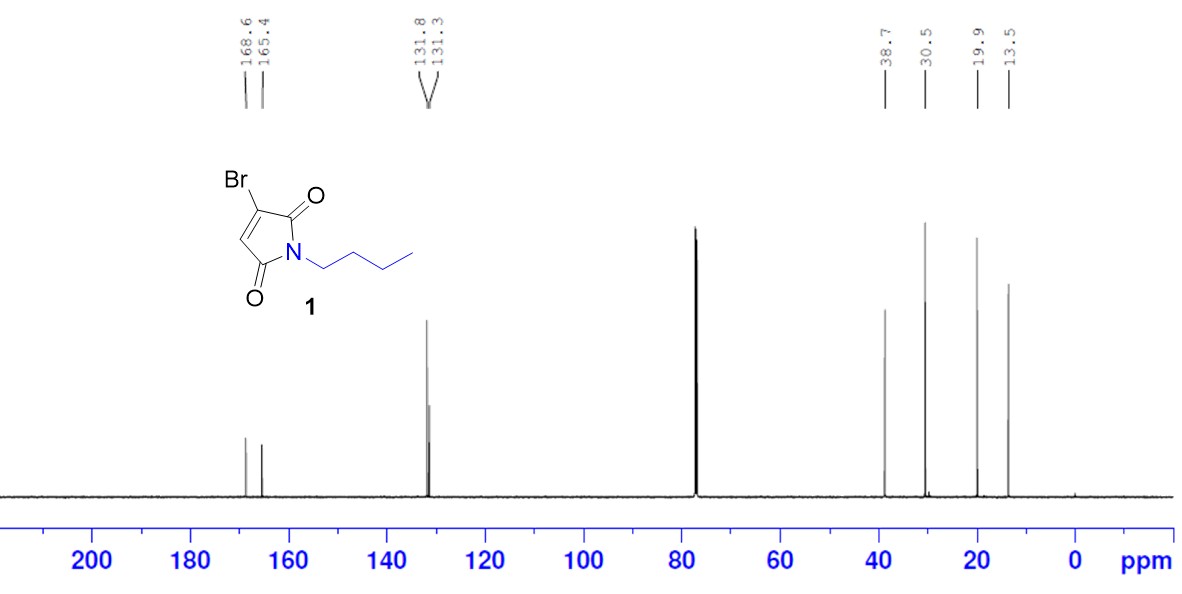
**

**S5**. HPLC for **2** (HPLC method: 60-95% B into A in 15 min at 220 nm)

**
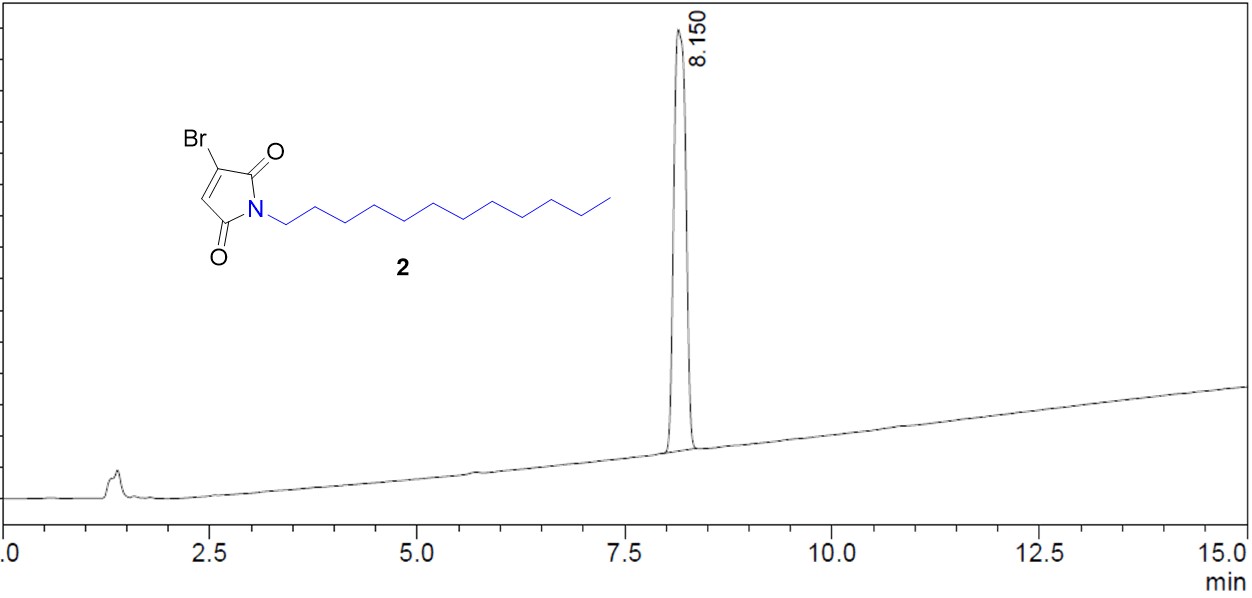
**

**S6.** ^1^H NMR for **2**

**
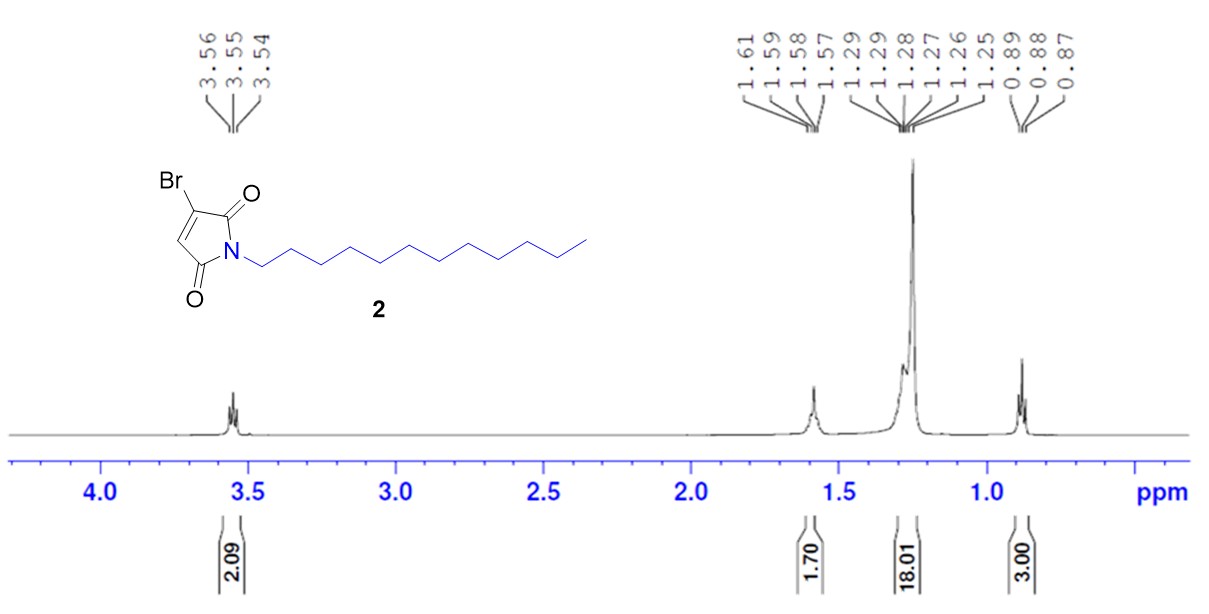
**

**S7.** ^13^C NMR for **2**

**
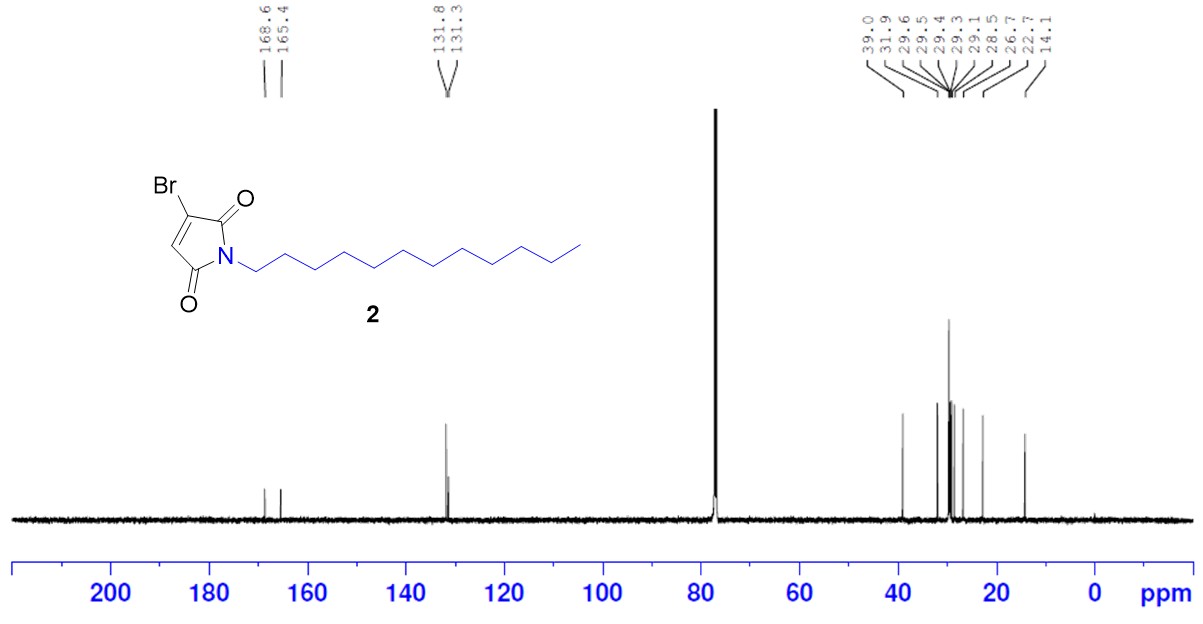
**

**S9**. HPLC for **3** (HPLC method: 5-95% B into A in 15 min at 220 nm)

**
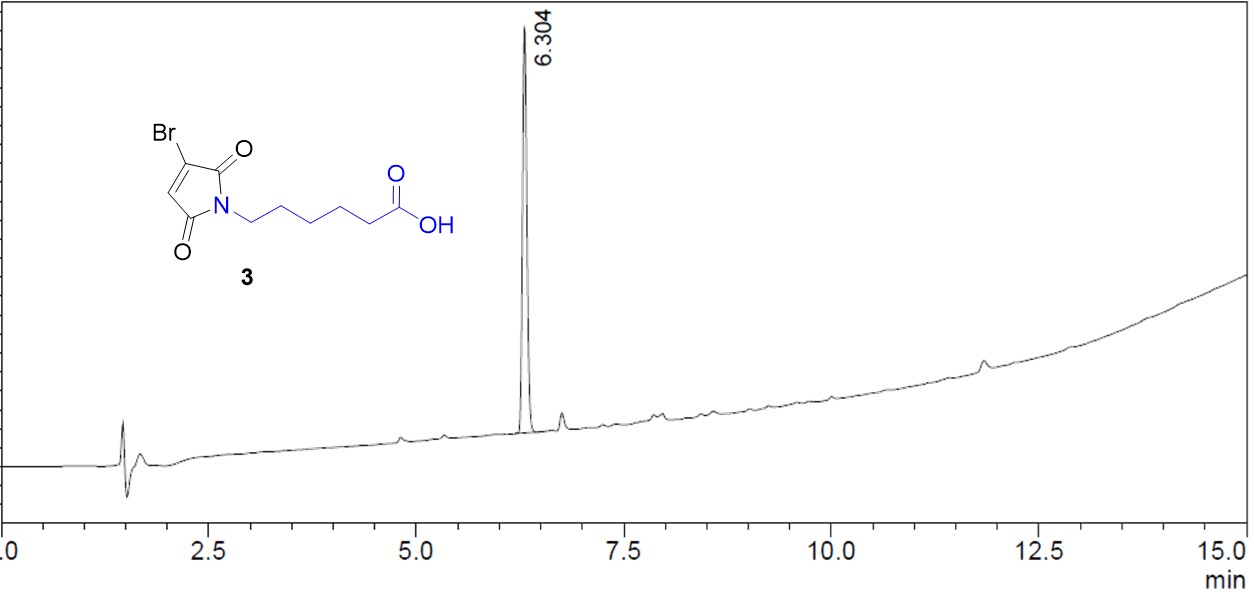
**

**S10.** ^1^H NMR for **3**

**
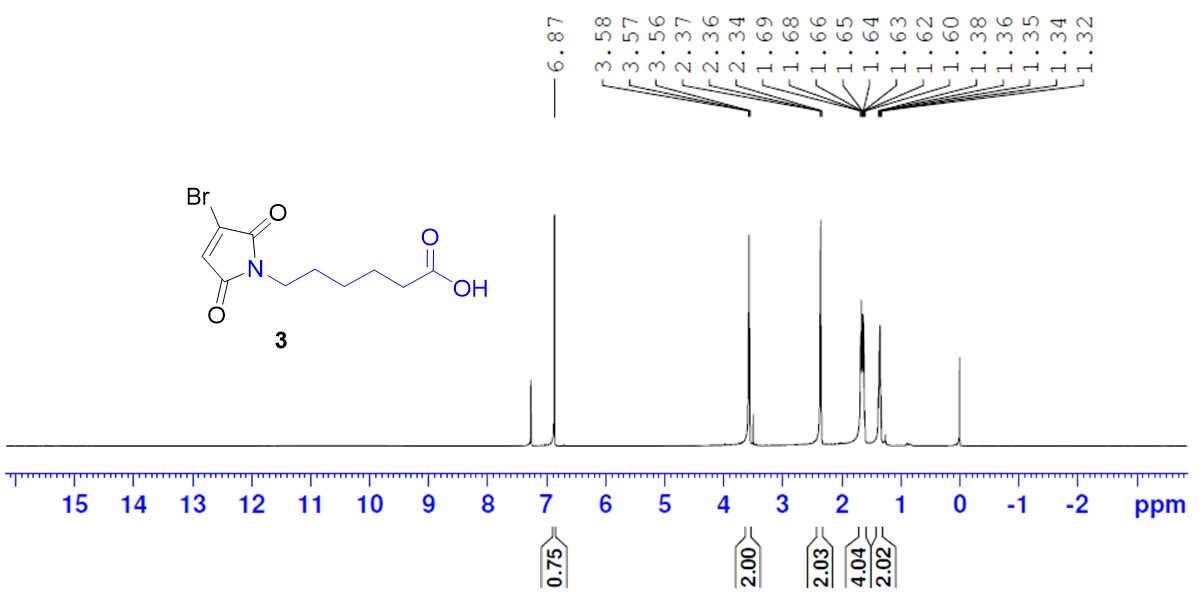
**

**S11.** ^13^C NMR for **3**

**
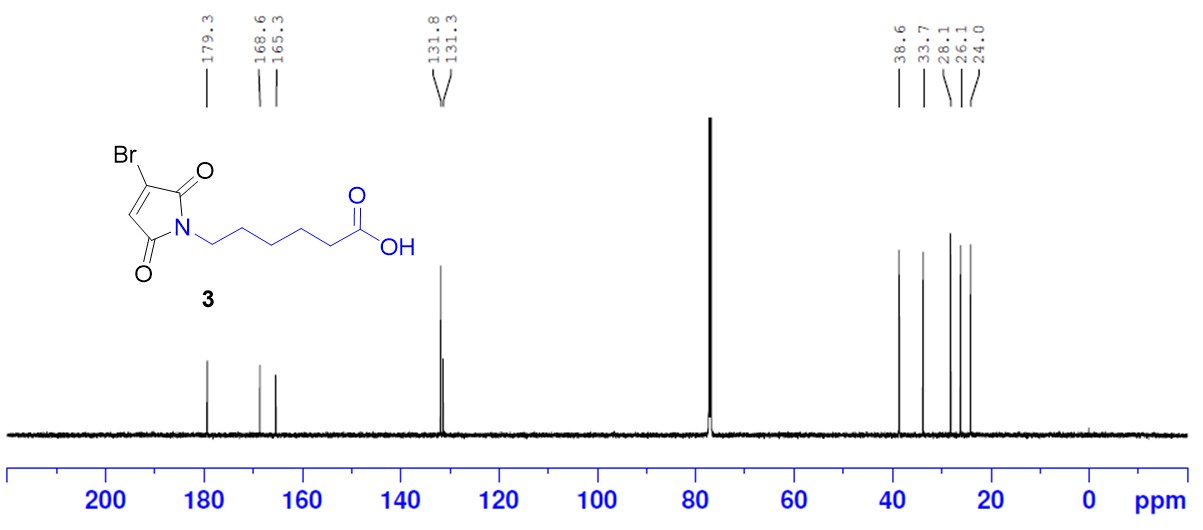
**

**S12.** HRMS for **3**


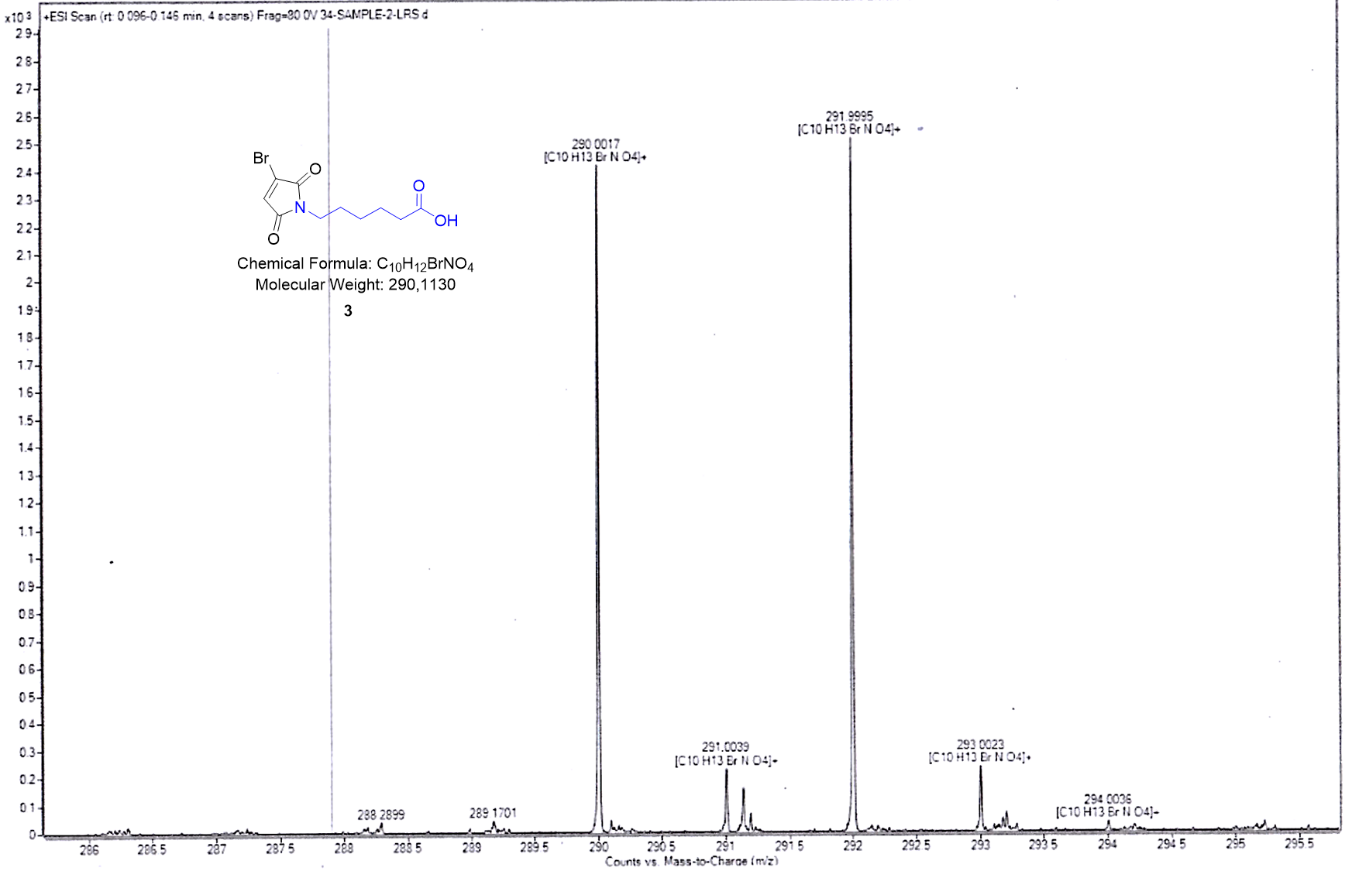


**S13**. HPLC for **4** (HPLC method: 5-95% B into A in 15 min at 220 nm)

**
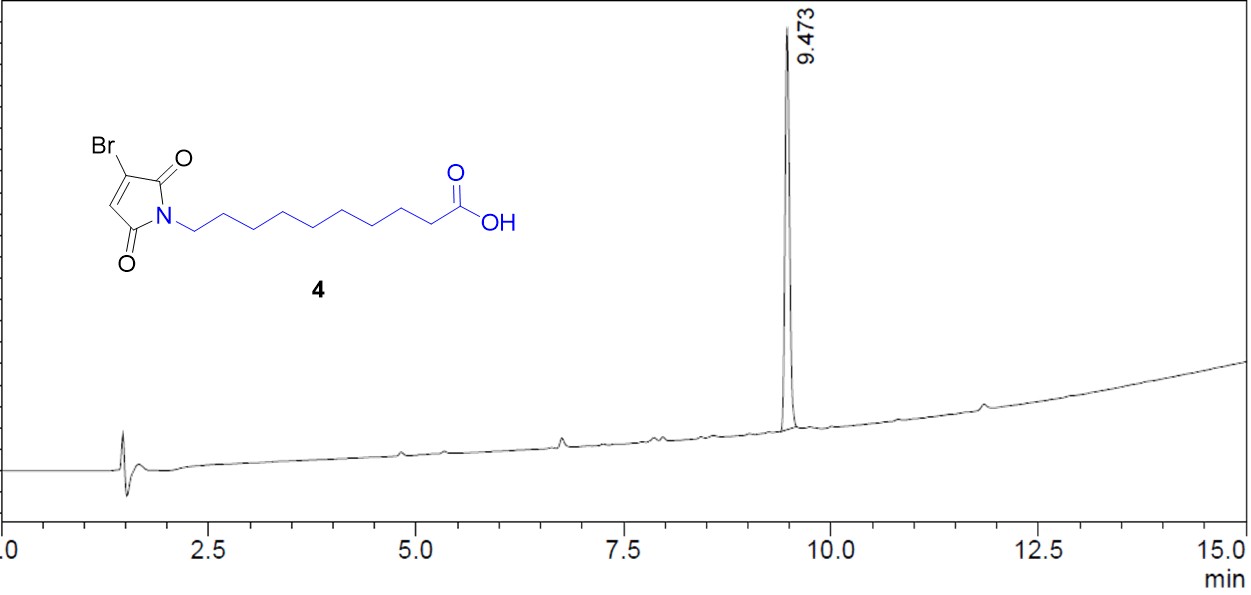
**

**S14.** ^1^H NMR for **4**

**
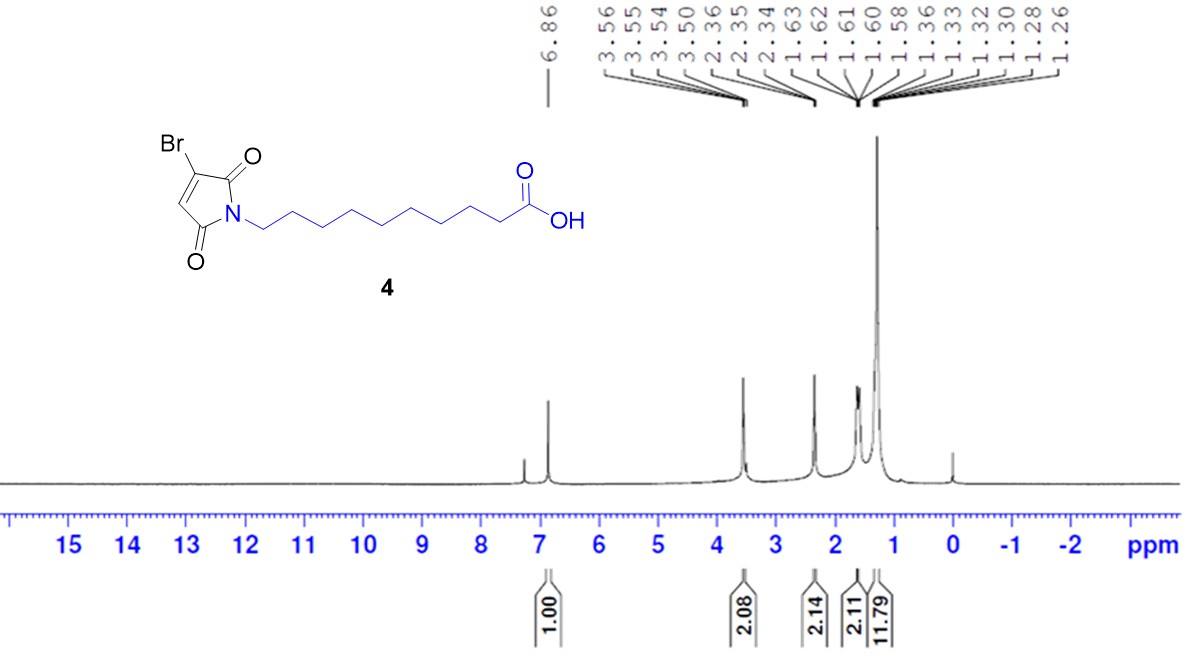
**

**S15.** ^13^C NMR for **4**

**
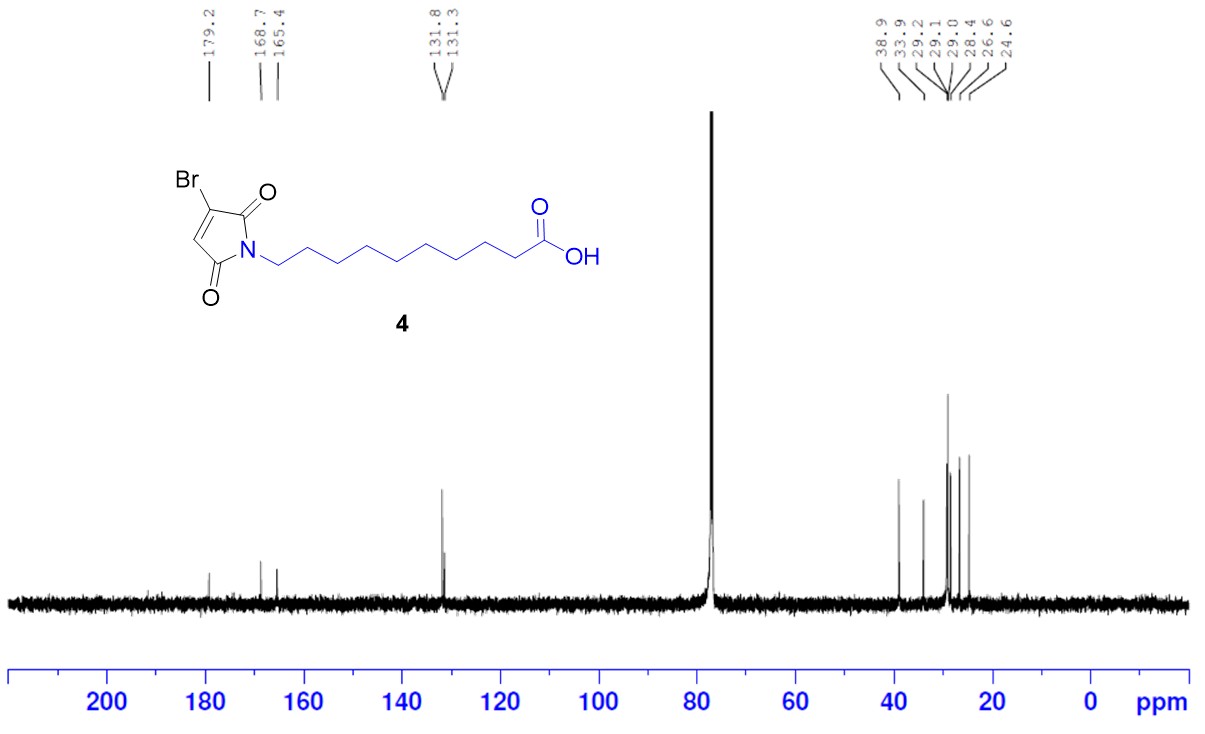
**

**S16.** HRMS for **4**


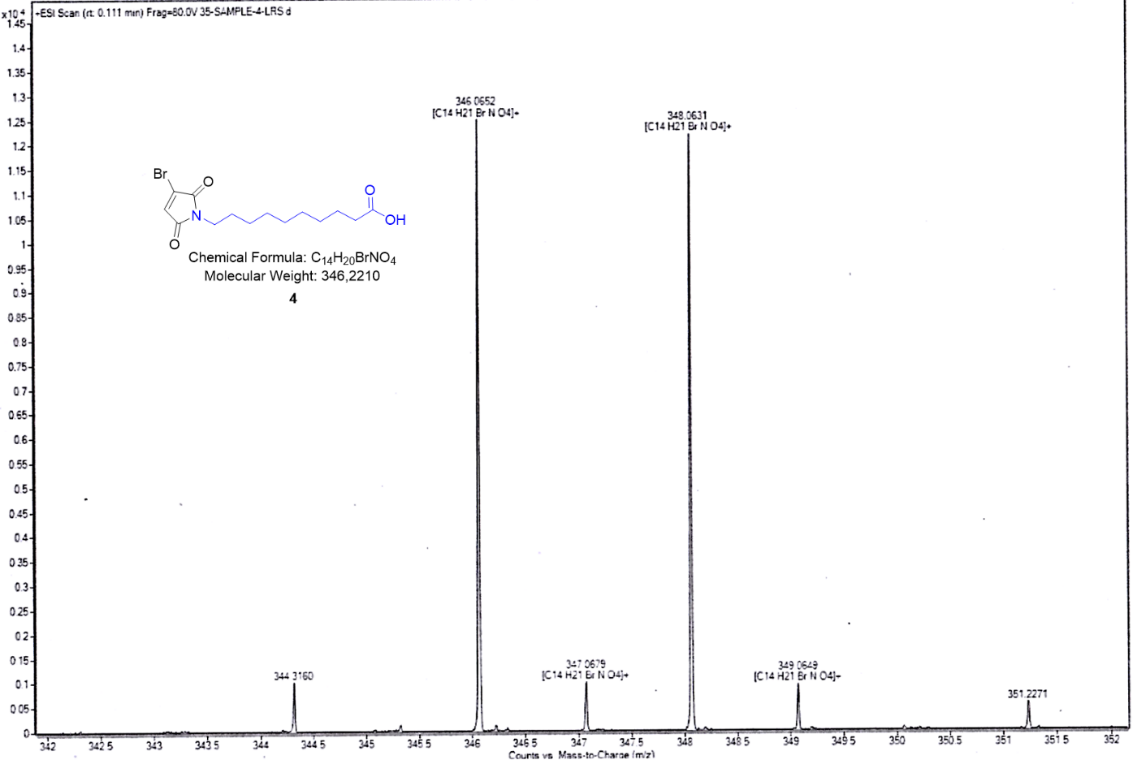


^1^H NMR for **5**

**
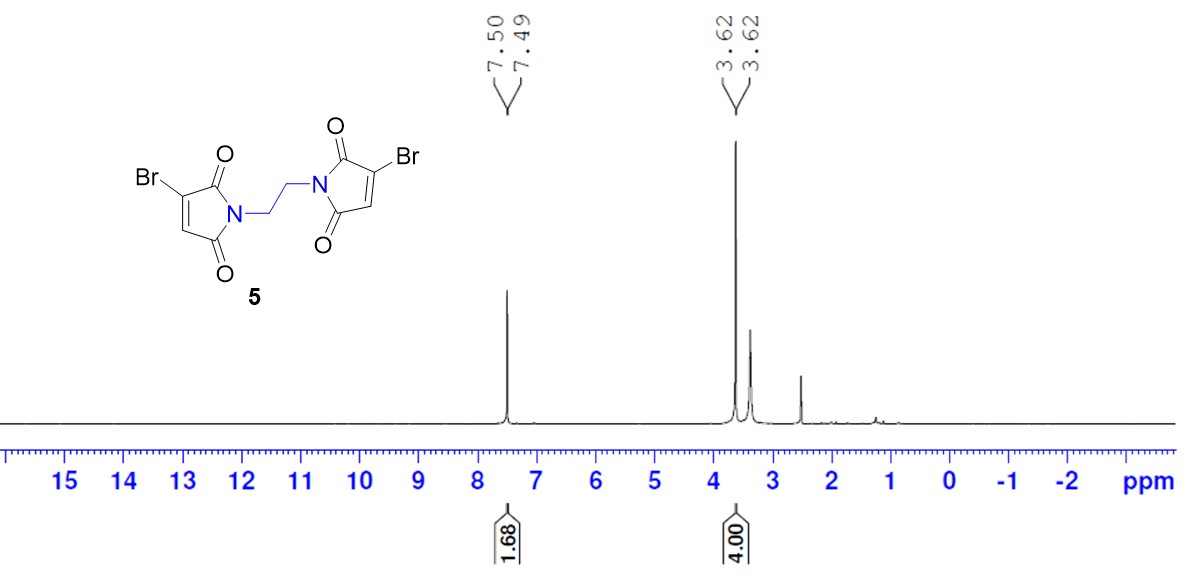
**

**S19.** ^13^C NMR for **5**

**
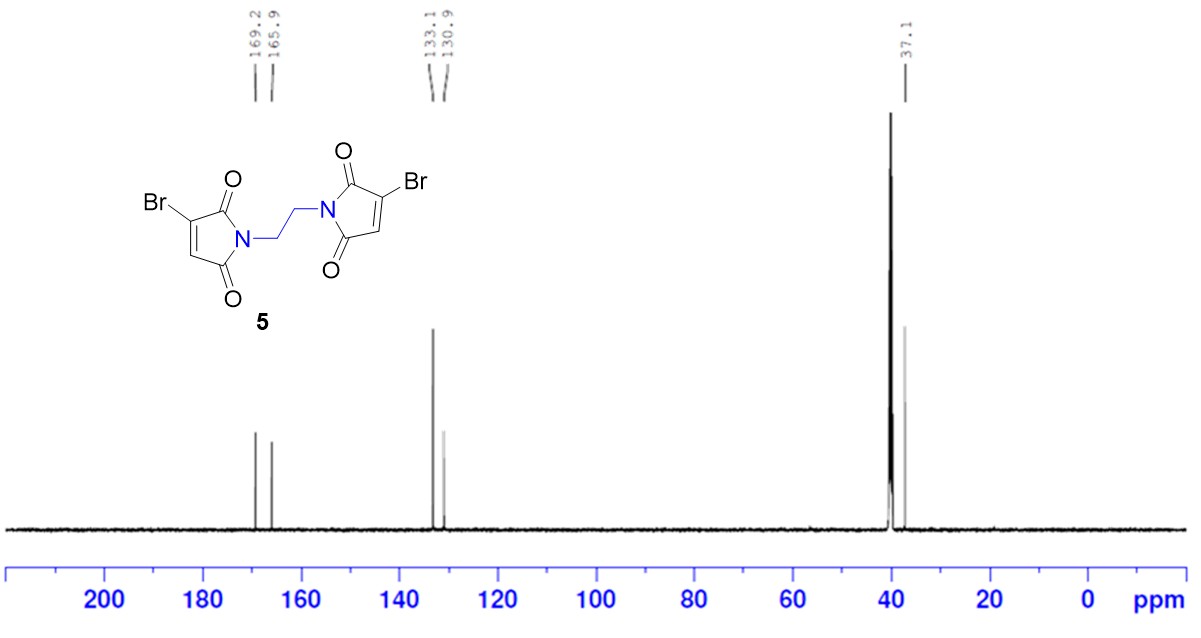
**

**S20.** HRMS for **5**


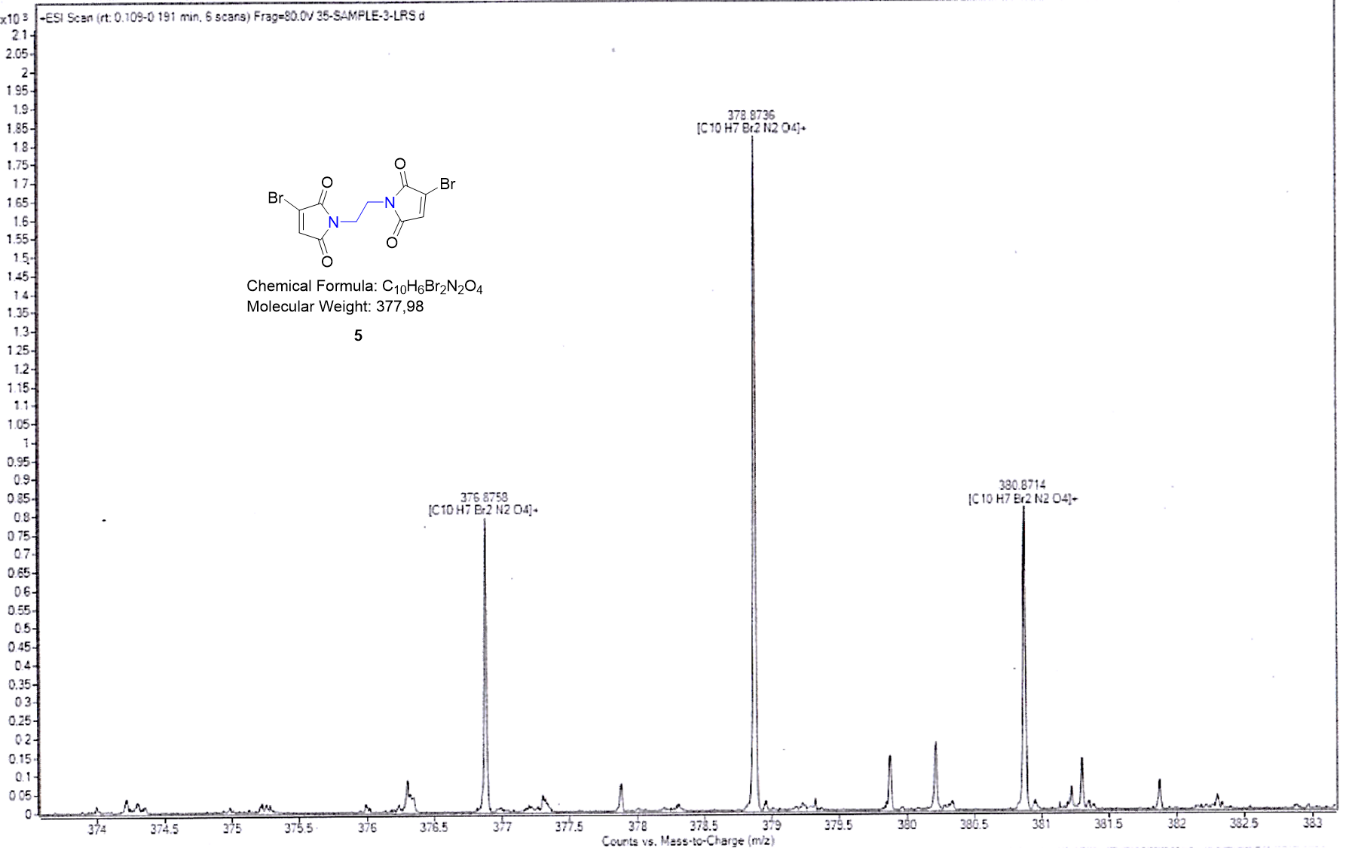

Supplement: Supplementary file 1 — Supplementary material and/or additional information [OPTIONAL] The supplementary Information containing the characterization data (HPLC, HRMS, 1H NMR and 13C NMR) is available free of charge. [file mmc1.docx]
